# Synthesis and Multi Scale Tribological Behavior of WC-Co/Nanodiamond Nanocomposites

**DOI:** 10.1038/s41598-017-07324-3

**Published:** 2017-08-01

**Authors:** Andy Nieto, Lin Jiang, Jaekang Kim, Dae-Eun Kim, Julie M. Schoenung

**Affiliations:** 1 Department of Chemical Engineering and Materials Science, University of California-Davis, Davis, CA 95616 USA; 2 Department of Chemical Engineering and Materials Science, University of California-Irvine, Irvine, CA 92697 USA; 3 Center for Nano-Wear, Yonsei University, Seoul, 120-749 Republic of Korea; 4 Department of Mechanical Engineering, Yonsei University, Seoul, 120-749 Republic of Korea

## Abstract

Nanodiamonds (ND) present a unique combination of desirable mechanical, functional, and chemical characteristics that are ideally suited for reinforcing and enhancing the wear resistance of carbide based materials. Tungsten carbide cobalt (WC-Co) matrix nanocomposites reinforced with varying amounts of ND (2 – 10 vol.%) were synthesized here by spark plasma sintering. The rapid thermal consolidation route enabled attainment of dense samples with a significant retention of the metastable diamond phase. NDs affected the microstructural evolution, chemistry, and mechanical properties of WC-Co. Macroscale reciprocating pin-on-disk tests were conducted to assess wear behavior under conditions relevant to service environments, e.g., high cycles and high contact pressure. Microscale tribological properties were assessed using microscratch tests in order to investigate the intrinsic effects of ND on the localized mechanical and tribological response of WC-Co-ND composites. The incorporation of 10 vol.% ND enhanced wear resistance at both the micro- and macroscale, by 28% and 35%, respectively.

## Introduction

Nanodiamonds (ND) have recently garnered attention for reinforcing metal matrix composites due to the outstanding mechanical^1, 2^, thermal^3^, and electrical properties^4^ of diamonds, alongside the unique structural characteristics of ND^5^. NDs feature a unique structure that consists of a 5–8 nm diamond core that is surrounded by a graphitic shell the thickness of which varies depending on the processing and purification methods utilized^6^. The thermal conductivity^7^, elastic modulus^8^, hardness^8, 9^, and wear resistance^7, 10, 11^ of copper and aluminum alloys have been improved by adding ND in amounts ranging from 0.5 vol.%^11^ to ~8.5 vol.%^8^. These early studies have explored relatively simple commercially pure alloys, and have not provided a mechanistic discussion on the effects of ND on properties such as hardness or wear resistance. The present study provides one of the first efforts to utilize ND as reinforcement for a complex ceramic based system, tungsten carbide cobalt (WC-Co).

WC-Co is a widely used ceramic based material with applications in aircraft breaks, drilling machinery, bearings, and other applications that require high wear and corrosion resistance. Conventional approaches to enhancing wear resistance consist of increasing carbide content, at the expense of the ductile Co binder. Recent efforts to enhance the wear resistance of WC-Co without adversely affecting the ductility include incorporating B_4_C particles^12–14^ and graphite or other free carbon sources^15, 16^. B_4_C is utilized because of its low density and its exceptionally high hardness, and has shown promising results in increasing the hardness and wear resistance of spark plasma sintered WC-Co-B_4_C composites^12–14^. The incorporation of free carbon is utilized to compensate for the decarburization and dissolution of carbide particles that occurs during synthesis. The solubility of tungsten into the cobalt binder, which results in a loss in ductility, is demonstrated to be substantially reduced by employing a carbon rich starting powder^16^. The present study seeks to enhance the wear resistance of WC-Co by combining these approaches. NDs are incorporated into WC-Co in order to enhance WC-Co wear resistance by providing both a higher hardness phase, as well as a carbon rich composition that will inhibit dissolution and the associated loss of ductility. Diamonds are the hardest known material, and the addition of nano carbon materials has recently shown promise for inhibiting decarburization in other carbide systems^17^.

In the present study, the effects of ND on the wear resistance and tribological behavior of a WC-Co are investigated, utilizing ND contents ranging from 2–10 vol.%. WC-Co-ND nanocomposites are synthesized by the spark plasma sintering technique. This technique utilizes a pulsing current to generate heat and thermal energy internally in the powder compact via joule heating. This technique has been demonstrated to reduce the sintering temperatures and dwell times necessary to consolidate ceramic based materials, while attaining superior properties as compared to conventional sintering routes^18^. The utilization of low temperatures and short sintering times is crucial in the current system in order to retain the metastable diamond phase during consolidation. A multi-scale approach is taken for evaluating the tribological behavior of the WC-Co-ND nanocomposites in order to distinguish intrinsic localized effects of ND from microstructural changes induced by the variable amounts of ND reinforcement.

## Results

### Nanocomposite Synthesis

**Figure 1:**
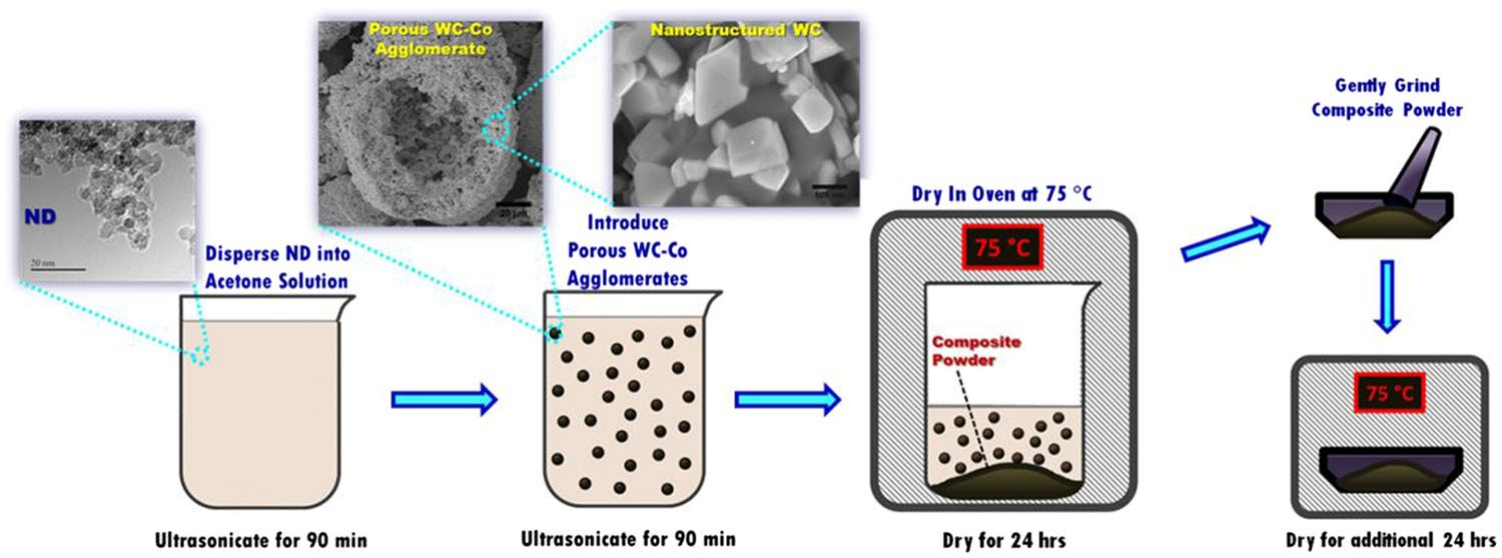
Schematic diagram of composite powder synthesis route including TEM and SEM of starting powders.

WC-Co and ND powders were mixed via a wet chemistry method (Fig. 1) because of its simplicity and the low temperatures utilized, which are ideal for processing the metastable ND phase. The porous nature of the starting WC-Co powder made the wet chemistry approach promising for ensuring ND particles were present within the WC-Co agglomerates. Acetone was chosen as the ultrasonication medium, as there are no known reactions between acetone and ND or WC-Co. ND powder was first ultrasonicated for 90 min in acetone. WC-Co particles were then introduced in the desired composition into the solution and ultrasonicated for an additional 90 min. The WC-Co/ND/acetone solution was then dried in an oven at 75 °C for 24 hrs. The powders were then crushed with a mortar and pestle, and then dried for an additional 24 hrs. Nanocomposite powders with volume fractions of 2 vol.% ND (WC-Co-2ND), 5 vol.% ND (WC-Co-5ND), and 10 vol.% ND (WC-Co-10ND) were synthesized. The nanocomposite powders were then consolidated into bulk form by spark plasma sintering (SPS). The rapid heating rates of SPS enabled rapid consolidation (<10 min), thereby enabling retention of the metastable nanodiamond phase.

### Microstructural Characterization

**Figure 2:**
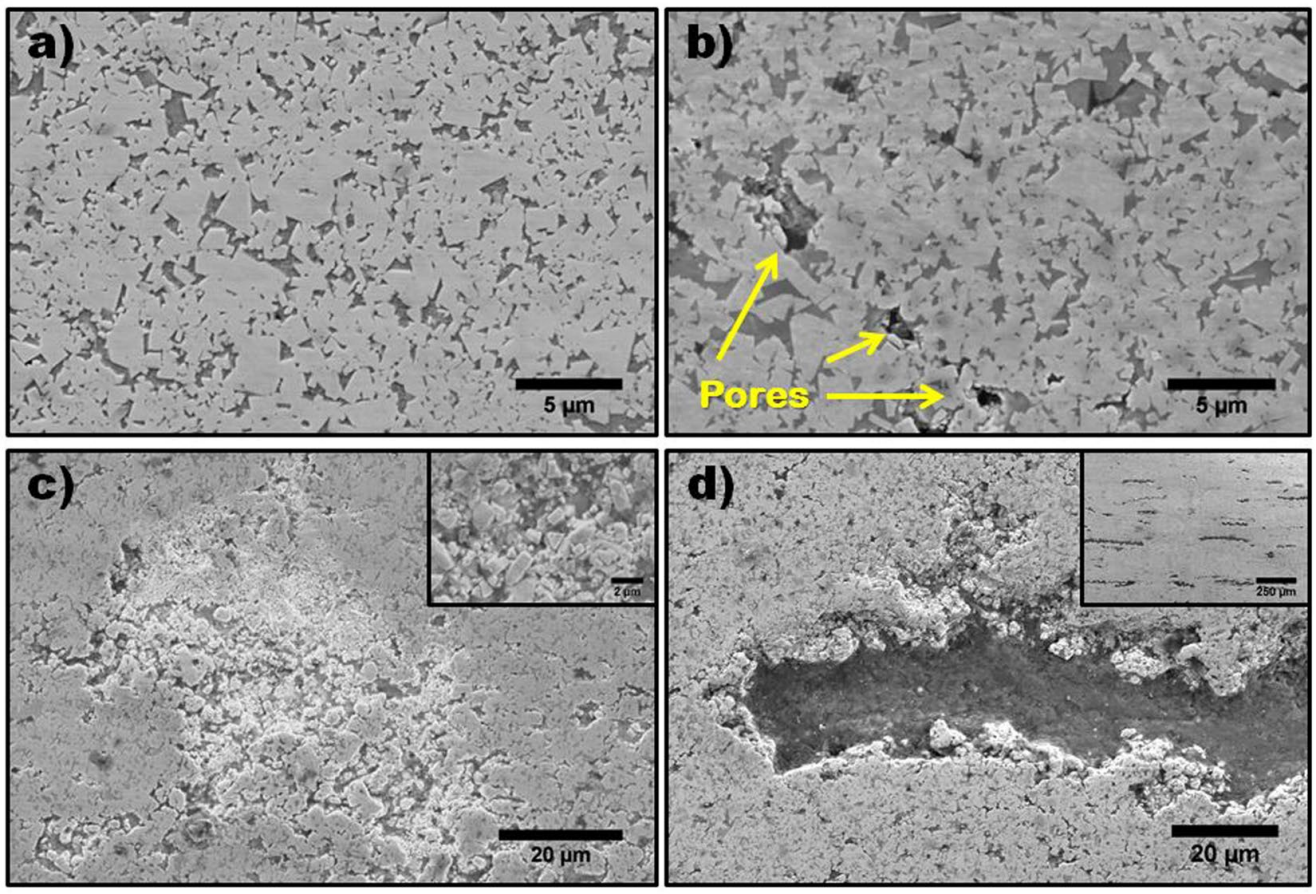
SEM of salient microstructural features in WC-Co and nanocomposite samples; (**a**) Dense WC-Co microstructure, (**b**) WC-Co-2ND exhibiting increased presence of micro pores, arrows denote examples of pores, (**c**) WC-Co-5ND showing poorly sintered regions with high porosity, Inset: high magnification image of pores in WC-Co-5ND, the region appears to have collapsed and bonding between WC and the Co binder is poor, (**d**) High aspect ratio porous region in WC-Co-10ND, Inset: Lower magnification image showing highly oriented nature and prevalence of high aspect ratio porous regions.

The quantification of relative density and average carbide particle size is presented in Table 1, and the typical microstructures associated with each consolidated composition are shown in the scanning electron micrographs (SEM) presented in Fig. 2. The average carbide particle size, which does not differ significantly among the varying compositions, is nearly twice that observed in the starting WC-Co powder, indicating that some degree of coalescence and grain growth occurred, despite the relatively low sintering temperatures and short sintering times. Differences among the compositions are observed, however, in the degree of densification. Densification is highest in WC-Co, and steadily decreases as the volume fraction of ND is increased. The image in Fig. 2a highlights that the WC-Co sample is mostly dense, with minimal signs of residual porosity present. Only a slight decrease (~2.5%) is observed with the incorporation of 2 vol.% ND, however, with the addition of 5 vol.% and 10 vol.% ND the decrease in densification was 6% and 6.9%, respectively. The microstructure of WC-Co-2ND exhibits an increased number of pores, typically 1–2 µm in diameter, throughout the structure, as shown in Fig. 2b. The porosity in the WC-Co-5ND and WC-Co-10ND samples exhibit distinctly different morphologies. The WC-Co-5ND sample exhibits significant porosity throughout its structure. The morphology of the porosity localized cracks, as shown in Fig. 2c. In addition, as shown in the inset of Fig. 2c, many carbide particles are poorly bound together, as there appear to be regions where pores have collapsed and adhesion with the Co binder is poor, indicating that bonds between WC and Co are weak. In contrast, the porosity in the WC-Co-10ND sample (see Fig. 2d) takes the form of elongated high aspect ratio pores that are aligned perpendicular to the applied pressure axis during sintering. The inset of Fig. 2d shows that these aligned pores are present throughout the sample, and can have lateral dimensions of over 200 µm, while their thickness is typically in the range of 10–20 µm. The remaining microstructure, including the regions surrounding the elongated high aspect ratio pores are largely dense, with porosity levels no higher than those observed in the microstructure of the WC-Co-2ND sample.

**Table 1:** Relative density and average WC particle size in WC-Co powders and consolidated materials.

Sample	Average Particle Size (µm)	Relative. Density (%)
WC-Co Powder	0.37 ± 0.14	—
WC-Co	0.68 ± 0.29	96.9
WC-Co-2ND	0.63 ± 0.25	94.4
WC-Co-5ND	0.70 ± 0.30	88.4
WC-Co-10ND	0.68 ± 0.34	87.5

**Figure 3:**
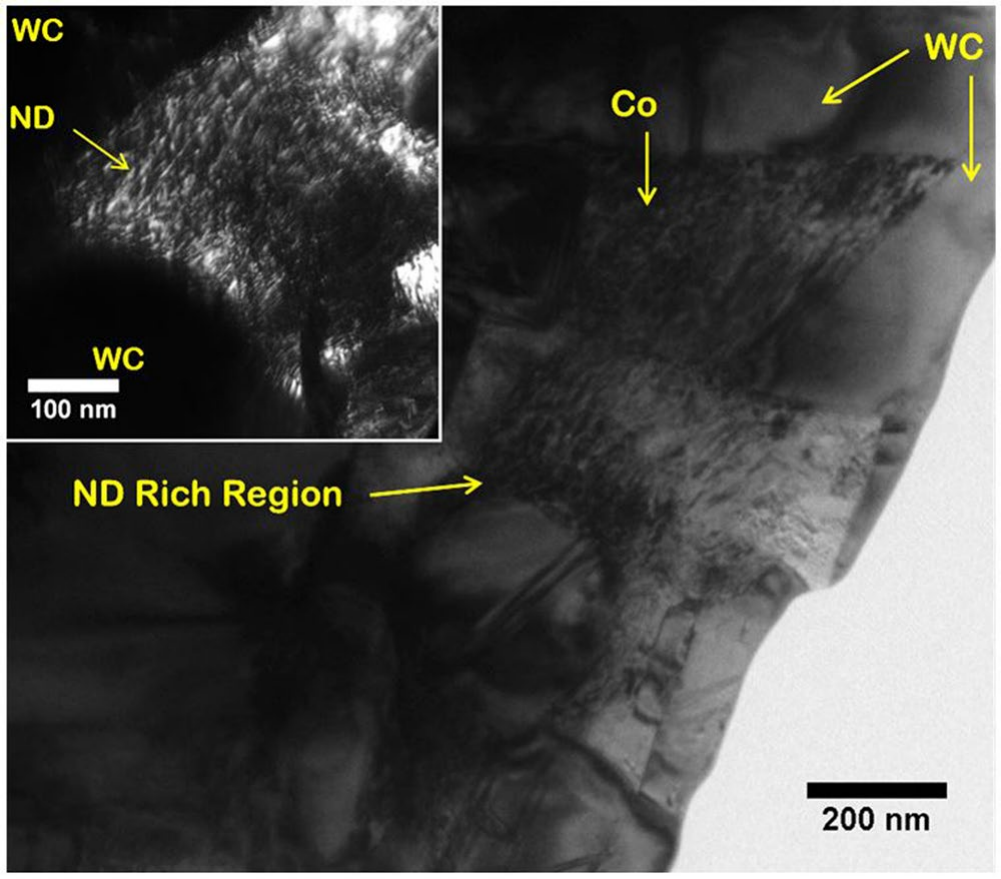
Bright field TEM of WC-Co-10ND sample, ND rich areas with adequate dispersion can be seen within the Co binder, inset: Dark field TEM image of ND rich region showing individual ~10 nm ND particles concentrated in Co binder, near a WC particle.

The presence of ND does not induce widespread compositional changes in the nanocomposites, as no additional phase formation is observed. X-ray diffraction (XRD) analysis, provided as supplementary information (Fig. S1), indicates that the primary phases are WC and Co in all four compositions. XRD is not sensitive enough to detect the ND phase. XPS analysis, provided as supplementary information Fig. S2), shows that the ND phase is indeed present in the nanocomposites, although some deterioration into other carbon phases (primarily graphite) occurred. This is expected due to the relatively high temperatures utilized during sintering, and the metastable nature of ND. The retention of the ND phase is greater in compositions with a higher volume fraction of ND. Transmission electron microscopy (TEM) of the WC-Co-10ND sample, shown in Fig. 3, provides further evidence of the retention of the ND phase within the nanocomposite. The TEM micrograph in Fig. 3 shows an ND rich region where NDs are uniformly distributed within the Co binder. The ND particles are expected to preferentially become embedded in the Co matrix during processing, due to the relative softness and higher ductility of the Co phase. The higher magnification dark field (DF) TEM image in the inset of Fig. 3 highlights that the ND particles are concentrated around the WC particles, but do not become embedded within the grains of the hard ceramic phase. The DF-TEM micrograph also shows that individual ND particles, mostly ~10 nm in diameter, are distributed within the ND rich region in the Co binder. The contrast in the DF-TEM micrograph confirms that these nanoparticles are compositionally different from Co and WC, indicating that they are indeed ND, and not precipitates.

### Macroscale and Microscale Tribological Properties

The tribological properties of WC-Co and nanocomposite samples at the macro and microscale are presented in Table 2 and Table 3, respectively. Macroscale wear resistance, as indicated by wear volume, is greatest for the WC-Co-10ND sample with a wear volume 35% lower than that for the WC-Co sample. The microscale wear resistance is also greatest for the WC-Co-10ND sample; the wear volume in this case is 28% lower than that for the WC-Co sample. The wear resistance of the WC-Co-2ND sample is not significantly different from that of WC-Co at both the micro and macroscales. The WC-Co-5ND has significantly lower wear resistance than the WC-Co sample during both macro and microscale wear testing. The wear volume for the WC-Co-5ND samples is 174% and 89% higher than that for the WC-Co sample at macro and microscales, respectively.

**Table 2:** Macroscale tribological properties of WC-Co and composite samples.

	Wear Volume (mm^3^)	Coefficient of Friction (COF)
WC-Co	0.23 ± 0.03	0.410 ± 0.017
WC-Co-2ND	0.21 ± 0.06	0.421 ± 0.008
WC-Co-5ND	0.63 ± 0.02	0.60 3 ± 0.006
WC-Co-10ND	0.15 ± 0.02	0.466 ± 0.023

**Table 3:** Microscale tribological properties of WC-Co and composite samples.

	Wear Volume (µm^3^)	Coefficient of Friction (COF)
WC-Co	44.0 ± 6.3	0.313 ± 0.005
WC-Co-2ND	45.2 ± 8.6	0.311 ± 0.004
WC-Co-5ND	83.3 ± 17.9	0.305 ± 0.008
WC-Co-10ND	31.6 ± 9.9	0.290 ± 0.008

Coefficients of friction (CoF) during macroscale testing are similar for the WC-Co and WC-Co-2ND samples. A slight increase in CoF is observed for the WC-Co-10ND sample due to the higher roughness induced by the samples porous structure. The wear tracks of the WC-Co-10ND sample had a roughness of R_a_ = 11 µm, while all other samples had a wear track roughness of R_a_ = 9 µm. The CoF is significantly higher for the WC-Co-5ND sample due to the much higher wear rate, which results in a higher contact area. At the microscale, porosity is not a significant factor due to the smaller volumes tested. The CoF for the WC-Co, WC-Co-2ND, and WC-Co-5ND samples are equivalent, while that for the WC-Co-10ND sample is slightly lower.

## Discussion

### Formation Mechanism and Effects of Elongated High Aspect Ratio Pores

**Figure 4:**
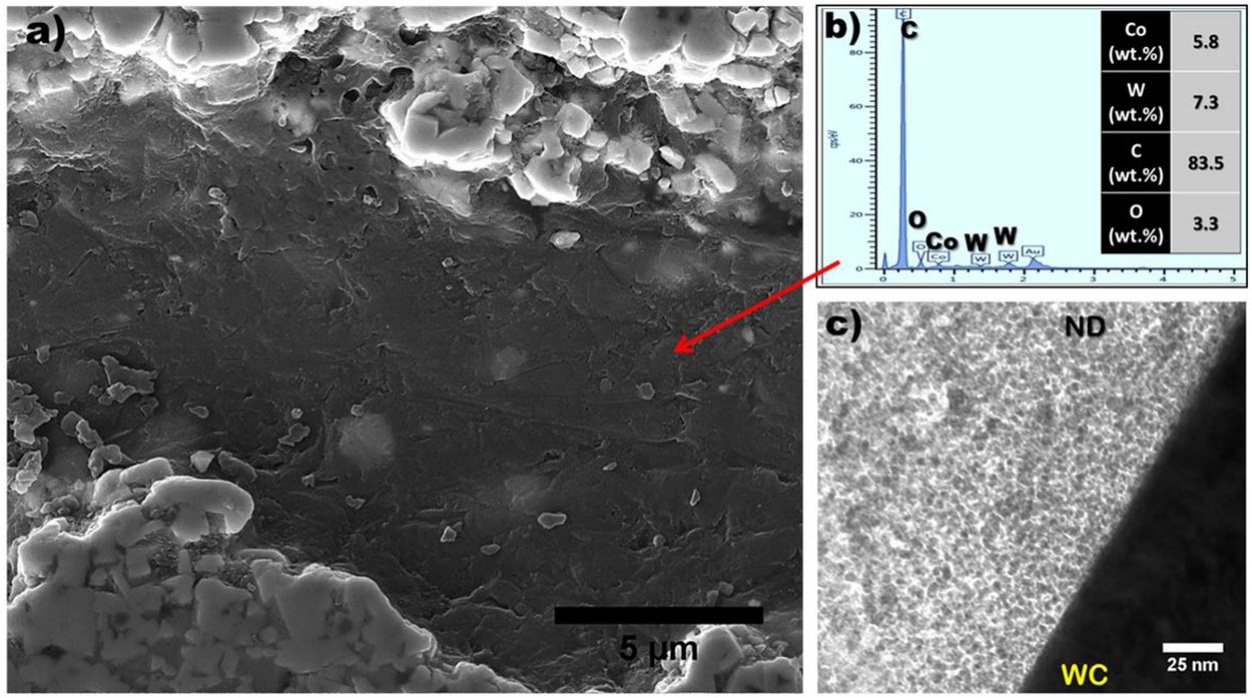
(**a**) SEM of interior of high aspect ratio pore in WC-Co-10ND sample, (**b**) EDS of pore walls indicating high carbon content, (**c**) TEM of smooth interface between agglomerated ND particles and WC particle, ND can be seen to be closely adhered to the WC grain.

**Figure 5:**
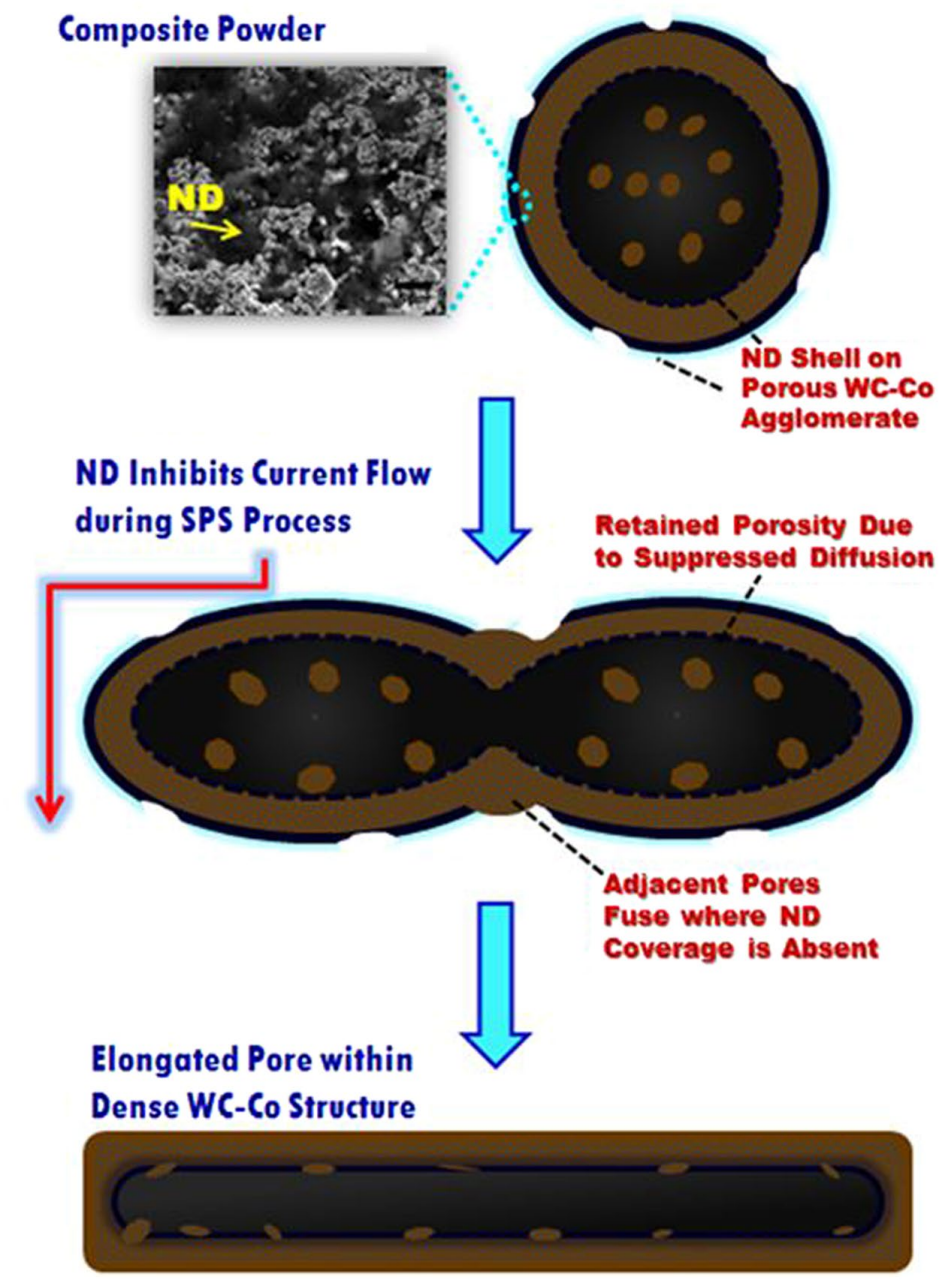
Schematic of formation mechanism of oriented high aspect ratio pores in WC-Co-10ND.

The significantly different tribological performance of the WC-Co-5ND and WC-Co-10ND samples can be attributed to the drastically different microstructures that evolve as a result of the high ND content, and hence it is important to understand the formation mechanism of the lamellar pores in WC-Co-10ND. SEM and XPS analysis of the pore interiors help shed light on the possible formation mechanism of these interesting features. Figure 4a is a high magnification SEM image of the interior of an elongated pore; the morphology and contrast indicate the pore interior is lined by a phase other than WC or Co. The interior of the pores are lined with a thin (at times electron transparent) layer of ND. Figure 4b presents EDS point analysis of the interior of the pore showing that the majority of the elemental composition is carbon, which is attributed to the ND phase. TEM analysis reveals that large ND agglomerates are found in the WC-Co-10ND structure, and that these agglomerates form a well adhered interface with WC, essentially forming a smooth shell on WC. The elongated high aspect ratio pores in the WC-Co-10ND sample are hence a direct result of the substantial presence of ND in this sample. The formation mechanism of these pores is also postulated to be related to the porous structure of the starting WC-Co powder, as shown schematically in Fig. 5. SEM analysis of the WC-Co-10ND powder revealed that NDs can form a continuous outer layer on the porous WC-Co spherical agglomerates. The porous nature of the WC-Co agglomerate and the use of a liquid solution powder processing route makes it feasible and likely that the ND liquid solution infiltrated the porous shell, enabling ND to also line the interior walls of the WC-Co porous agglomerate. The high electrical resistance of ND would act as a current barrier during SPS processing, which relies on the flow of current to generate internal joule heating within the powder.

Electrically insulating phases are often used in SPS to direct current flow, albeit, at a meso or macroscale^19^. As the SPS processing of the nanocomposite powder commences, the porous WC-Co-ND agglomerates deform, and the ND films on the surface inhibit current flow, which thereby hinders heat transfer and solid state diffusion. As indicated in the SEM of the powder and the schematic of the powder in Fig. 5, the ND shells are not completely homogenous on the agglomerate surface, but rather there are gaps in ND coverage. Adjacent spherical agglomerates may fuse together when surfaces with limited ND content are in contact, as current can flow, enabling necking and sintering to occur. As sintering progresses, these deformed agglomerates become highly elongated pores with lateral dimensions that vary from tens of microns to over 200 µm. The pores are aligned perpendicular to the sintering axis as their formation is dependent on the high applied pressures during SPS. The thickness of the elongated pores is typically 10–20 µm, which is consistent with the approximate size of the starting spherical agglomerates (15–45 µm). The lateral dimensions vary depending on the number of adjacent agglomerates that fuse together. These elongated high aspect ratio pores do not evolve in other nanocomposite compositions because the ND coverage of the WC-Co agglomerates is not sufficient to provide a significant current barrier. Likewise, higher ND contents beyond 10 vol.% would be expected to lead to an equivalent or increased evolution of such elongated high aspect ratio pores.

The significantly different pore structures lead to significantly different responses to tribological loadings, leading to the drastic differences in wear resistance observed for the WC-Co-5ND and WC-Co-10ND samples. The orientation of the elongated high aspect ratio pores perpendicular to the sintering axis, as well as the applied loading direction, ensure that these pores will not facilitate crack initiation or propagation. Instead compressive forces will tend to compress or close the pores, thereby inhibiting crack growth. This compressive deformation of pores provides an enhanced strain tolerance effect, analogous to that observed due to columnar pores in thermal barrier coatings^20^. The deformation of pores, so long as crack initiation/propagation does not occur, enables strains to be relieved, which lowers the effective normal loading and thereby provides a mechanical damping effect^20–22^. Deformation of pores essentially consumes or dissipates strain energy that would otherwise be released through plastic deformation or crack formation/propagation, both of which lead to greater wear of the material.

This proposed strain tolerance effect is also consistent with established relations that correlate lower bulk elastic modulus with greater wear resistance^23, 24^. The elongated pores in the WC-Co-10ND effectively decrease the elastic modulus of the bulk sintered compact due to the inability of the pore volume to bear loads. The high aspect ratio of the pores is also beneficial as it increases the cross-sectional area of the pores relative to the applied normal loading direction. An increased cross-section enables the damping effect to occur over larger areas at a time, which is an advantage during dynamic tribological loads where the applied normal load is not stationary. The geometry of the porosity in the WC-Co-10ND is hence the key factor that enables it to have a beneficial effect, due to the orientation of the pores, which is not conducive to crack initiation.

In contrast, the high porosity content in WC-Co-5ND is not beneficial as pores exhibit random and irregular geometries and often have pre-existing cracks in their vicinity. The absence of a high aspect ratio or preferred orientation of these pores makes the porosity unfavorable for resisting normal or shear loadings induced by reciprocating contact sliding. Instead compressive loads may elongate pores and lead to crack initiation and/or propagation of existing cracks. These pores and pre-existing cracks present in WC-Co-5ND hence promote crack initiation and propagation that accelerates wear, and any strain tolerance effect in the WC-Co-5ND sample is insignificant.

### Effect of ND on Tribofilm Formation

**Figure 6:**
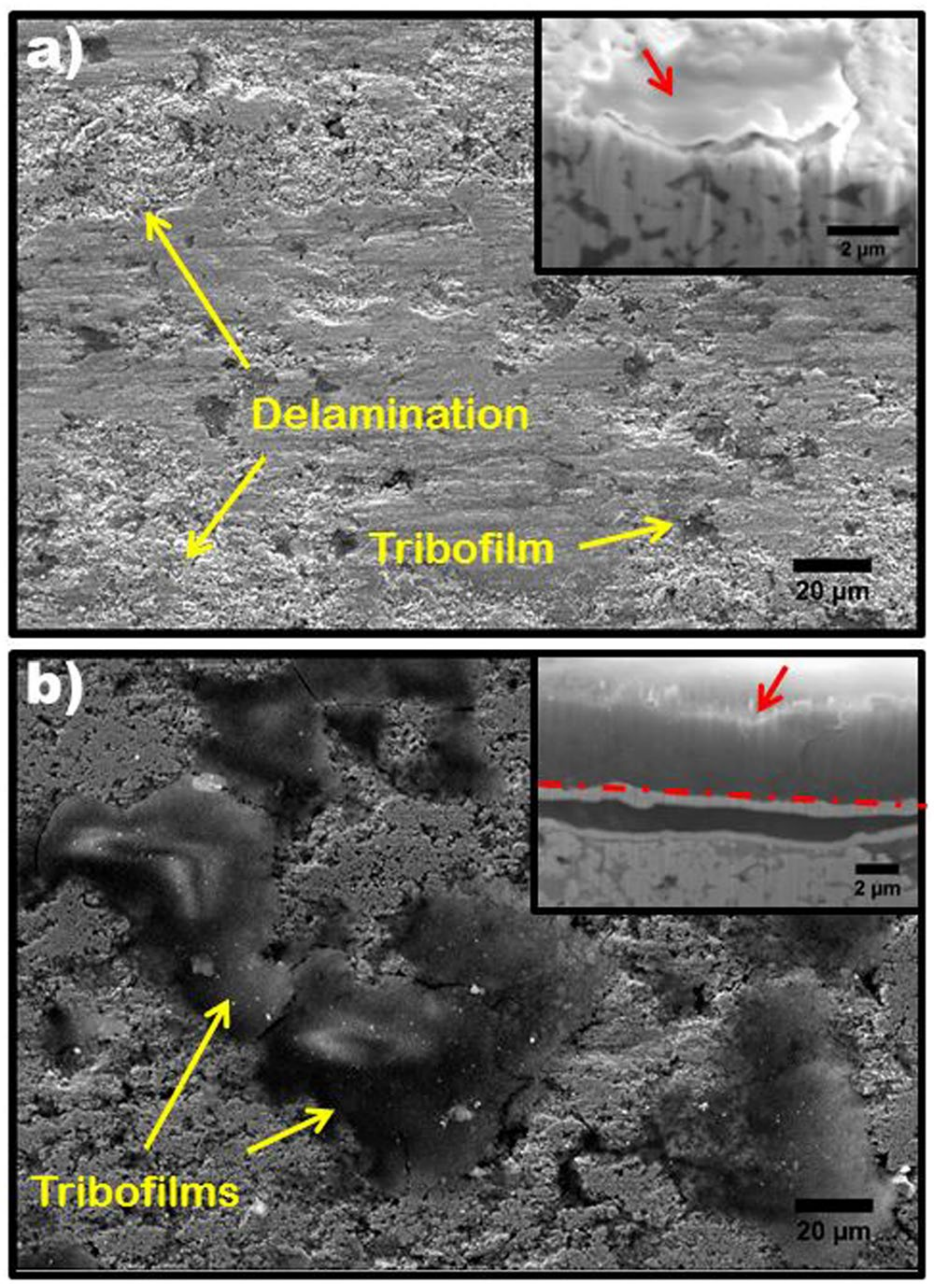
SEM of macro scale wear tracks; (**a**) WC-Co exhibits signs of delamination and tribofilms are typically smaller than 20 µm, inset: FIB milled cross-section showing thickness of tribolayers in WC-Co is < 300 nm, (**b**) WC-Co-ND wear track shows large (>50 µm) tribofilms and no signs of delamination, inset: FIB cross section shows thickness of tribofilms is 4–5 µm. Red arrows denote tribofilms in insets.

Tribofilm formation during wear is dependent on the heat generated from friction to catalyze the tribochemical reaction. The high thermal conductivity of ND increases heat transfer to the sample being worn and thereby promotes a robust tribofilm formation. The stark differences in the tribofilms developed in WC-Co and WC-Co-10ND sample can be observed in the track morphologies presented in Fig. 6. The chemical composition of this tribofilm is confirmed to be SiO_2_, using EDS and XPS analysis, presented as supplementary information (Fig. S3), as well as in our previous studies on WC-Co-ND composite coatings^25^. Figure 6 demonstrates that the coverage of the wear track by a silica tribofilm is significantly greater in the composite sample, and this contributes to a less severe wear response. The scant tribofilm formation in WC-Co does not provide significant protection and the delamination is observed to readily occur on the wear track, contributing to higher roughness and wear rates. In addition, FIB sectioning of the tribofilms, provided as insets in Fig. 6, show that the tribofilms formed on the WC-Co-10ND wear track are 4–5 µm thick, while those formed on WC-Co are less than 300 nm thick. The hard silica film also preferentially forms on pores, due to the need for Si_3_N_4_ debris to accumulate, before it oxidizes to form the silica tribofilm. The exposure of the oriented high aspect ratio pores in WC-Co-10ND during wear is thereby mitigated by the formation of thick tribofilms at the pore sites, essentially creating a self-healing effect when pores become exposed. The formation of this tribofilm is believed to be the primary factor that yields the higher wear resistance in the WC-Co-2ND samples, as this sample does not appear to benefit from other ND induced microstructural or chemical features.

### ND Induced Inhibition of Carbide Dissolution

The impact of ND on the chemistry of the WC-Co and composite systems can be understood from the EDS point analysis conducted on WC particles and the Co binder, which are tabulated in Tables 4 and 5, respectively. The WC-Co sample has a considerably lower amount of W after SPS processing, as compared to the starting powder. This indicates that excess W has left the carbide particles and has been solutionized into the Co binder. This is supported by Table 5, which indicates that the amount of W found within the Co binder is much higher in the sintered WC-Co sample, as compared to the as-received sample. This dissolution of WC into the Co binder is significantly inhibited in the composite samples. The amount of W found in the Co binder in the composite samples is dramatically lower, and the amount of W in the WC particles steadily increases with increasing ND content. This evidence indicates that the presence of ND does indeed provide a carbon-rich environment that inhibits dissolution and decarburization of WC particles, as dissolution of W into the binder is only possible when there is excess W. The excess carbon also makes it unfavorable for decarburization to occur even at high temperatures (up to ~2750 °C), as the thermodynamically stable composition will be ‘WC + C’^26^.

**Table 4:** EDS point analysis of WC particle region in powder and SPS consolidated samples.

Carbide Regions	Co (wt.%)	W (wt.%)	C (wt.%)	O (wt.%)
WC-Co Powder	0.8	93.6	5.6	0.0
WC-Co	2.7	86.5	9.5	1.3
WC-Co-2ND	2.6	88.8	8.0	0.5
WC-Co-5ND	2.9	89.2	7.8	0.1
WC-Co-10ND	1.3	91.1	6.9	0.1

**Table 5:** EDS point analysis of Co binder region in SPS consolidated samples.

Co Binder Regions	Co (wt.%)	W (wt.%)	C (wt.%)	O (wt.%)
WC-Co	74.0	18.7	5.6	0.9
WC-Co-2ND	87.7	2.7	8.5	1.0
WC-Co-5ND	91.3	3.8	4.7	0.2
WC-Co-10ND	89.4	4.7	5.7	0.2

**Figure 7:**
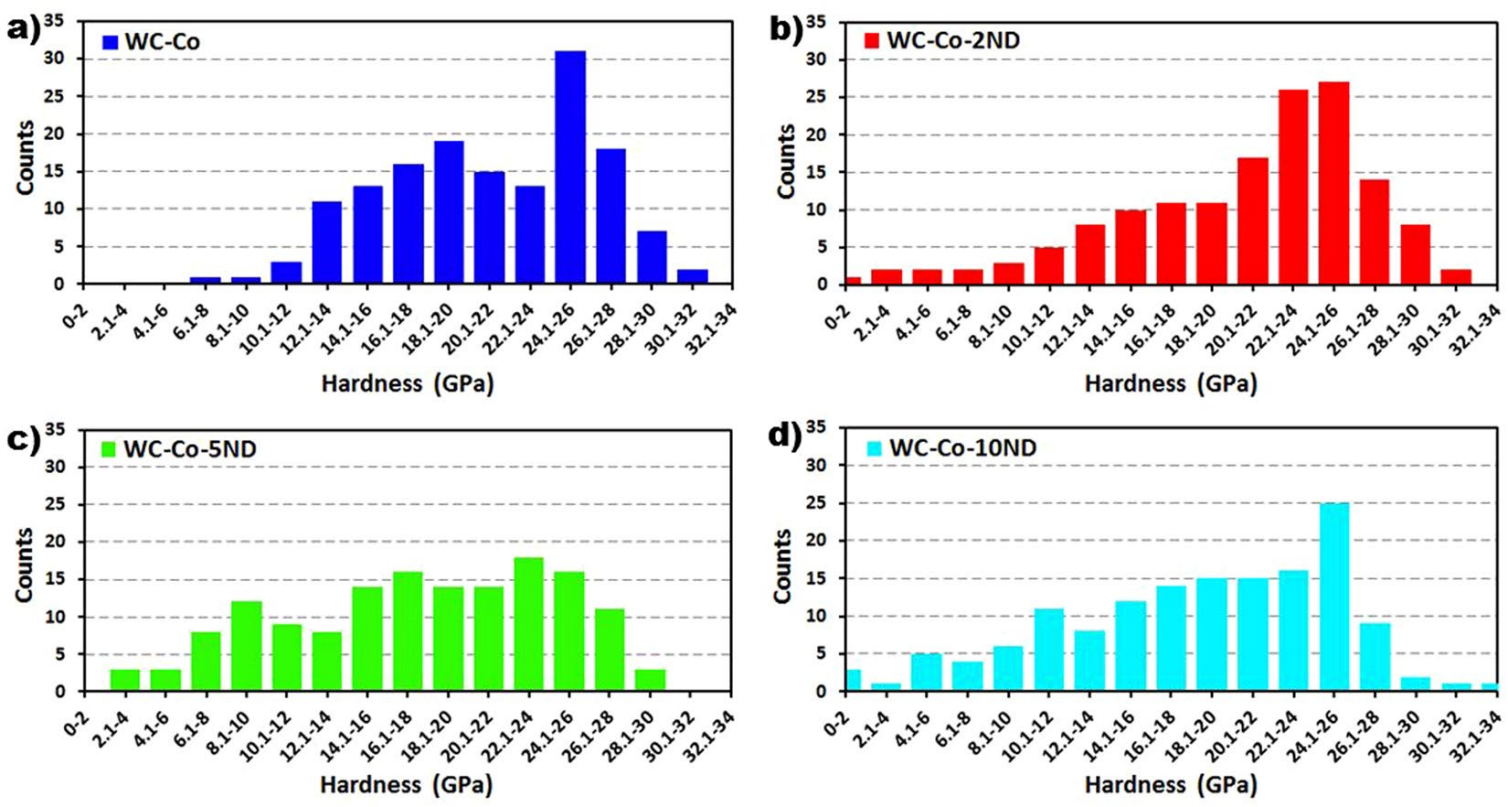
Distributions in nanohardness measured via nanoindentation; (**a**) WC-Co, (**b**) WC-Co-2ND, (**c**) WC-Co-5ND, (**d**) WC-Co-10ND.

**Figure 8:**
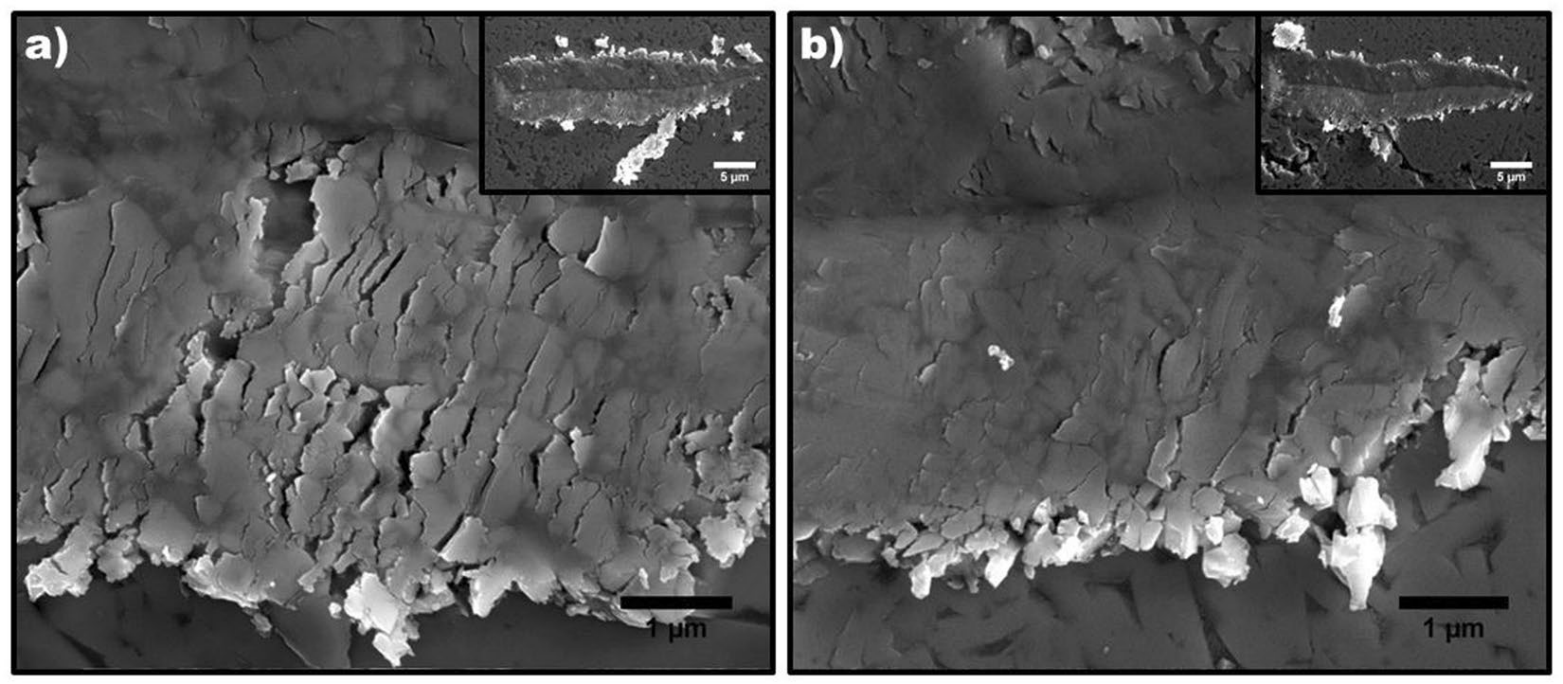
SEM of micro scratch grooves; (**a**) WC-Co scratch groove exhibits significant cracking, indicative of an embrittled binder, inset: lower magnification image of a WC-Co scratch groove exhibiting cracking and large debris particle, (**b**) WC-Co-10ND scratch groove does not exhibit any significant cracking, groove is smooth indicative of a ductile binder, inset: lower magnification image of a WC-Co-10ND scratch groove showing significantly reduced cracking and debris.

Evidence for inhibition of W dissolution into the Co binder is also supported by the nanohardness distribution data, shown in Fig. 7. The sintered WC-Co exhibits very few nanohardness data point values below 10 GPa, which would be indicative of a pure Co binder. In fact, EDS analysis indicates a significant amount of W is present within the Co binder, which will increase the strength of the binder at the expense of ductility. In contrast, the composite samples with high ND content exhibit a significantly higher proportion of nanohardness data point values below 10 GPa, indicating the presence of the softer and more ductile Co binder phase. These nanoscale mechanical properties explain the behavior observed during microscale wear. The most dramatic effect can be seen upon inspection of the scratch grooves of the WC-Co and WC-Co-10ND samples, shown in Fig. 8. The wear groove of WC-Co exhibits severe cracking and fairly large debris. In contrast, the WC-Co-10ND scratch groove is mostly smooth, indicative of a ductile wear response, with only small debris particles. This ductile wear response at the microscale is attributed to the retention of the ductile Co binder in the samples with high ND content. It should be noted that the WC-Co-5ND sample does not appear to benefit from the increased presence of a ductile binder, due to the simultaneous softening of the overall material due to the significant presence of homogeneous porosity. The nanohardness distributions reveal a significantly lower proportion of hardness values above 22 GPa, as compared to all other samples. The elastic modulus distributions exhibit similar trends, such as a higher proportion of values below 300 GPa in high ND content samples, and are provided as supplementary information (Fig. S4).

## Conclusions

To summarize, the addition of 2 vol.% ND leads to a slight increase in wear resistance at the macroscale due to the enhanced formation of a protective tribofilm. At the microscale, the inhibition of carbide dissolution does not translate to different mechanical or tribological properties, as compared to WC-Co. The addition of 5 vol. % ND leads to widespread porosity, which yields lower wear resistance at both micro and macroscales. The addition of 10 vol.% ND enhances wear resistance at the microscale due to the retention of a ductile binder. At the macroscale, the WC-Co-10ND sample also benefits from increased formation of a protective tribofilm, and an oriented and high aspect ratio pore structure that may enhance strain tolerance, which together yield an enhancement in wear resistance. Thus, the presence of ND has a complex effect on WC-Co, as ND can affect chemistry, microstructural evolution, and mechanical behavior. The utilization of high ND volume filler (10 vol.%) is demonstrated to provide increased wear resistance at both micro and macroscales, while reducing overall weight due to the inherent porosity and lower density of the ND phase. These nanocomposites could yield energy savings and performance enhancements in automotive, aerospace, and industrial applications that require wear resistant materials with a minimal weight penalty. The use of ND together with the SPS technique may be suitable for controlling and developing microstructural architectures with tailored mechanical and functional properties in other ceramic and metallic systems.

## Materials and Methods

### Materials

The starting ND powder was procured from Nanostructured & Amorphous Materials, Inc. (Synthesized Nanodiamond #1321JGY, Houston, TX). Nanodiamonds were synthesized by the detonation explosion synthesis method and had a purity of 98% diamond, as specified by the manufacturer. The ND powder had a specific surface area of 282.2 m^2^/g, and the primary impurities included Fe (0.062 wt.%) and Si (0.014 wt.%), and all other impurities were less than 0.01 wt.%, as specified by the manufacturer. A previous study reported the true density of ND powder to be 3.22 g/cm^3^, as determined using the helium pycnometry technique^11^. The starting WC-Co powder was procured from Inframat Advanced Materials (Infralloy S7412, Manchester, CT) and had a composition of 88 wt.% tungsten carbide and 12 wt.% cobalt, which corresponds to a theoretical density of 14.82 g/cm^3^. The starting WC-Co powder consisted of 15–45 µm agglomerates that were made up of nanostructured WC particles with a size range of 370 ± 140 nm, embedded in a Co binder.

Bulk composite samples were obtained by consolidating the mixed nanocomposite powders using a DR. SINTER SPS-825 S (Fugi Electronic Industrial Co., Kawasaki, Japan) spark plasma sintering furnace. Nanocomposite powders were packed into 20 mm graphite dies lined with graphite foil. Samples were sintered using a dwell pressure of 90 MPa, a dwell temperature of 1050 °C, a dwell time of 5 min, and a heating rate of 300 °C/min. The furnace chamber was held in vacuum throughout the sintering process, with a residual pressure of ~3–4 Pa.

### Mechanical and Tribological Characterization

Sample cross sections were mounted and ground using progressively finer silicon carbide abrasive papers, down to a grit size of 600; and then polished using progressively finer diamond slurries, with the final polishing step utilizing a slurry with 50 nm diamond particles. Mechanical properties were evaluated via nanoindentation to measure the localized distributions of elastic modulus and nanohardness in the multi-phase nanocomposites. Nanoindentation tests were conducted using an MTS Nano Indenter XP using a Berkovich tip. The tip area calibration was conducted using a standard quartz sample with a known reduced elastic modulus of 69.6 GPa. The nanoindentation load cycle consisted of a 10 second ramp to the maximum load of 1 mN, with a holding time of 3 seconds, followed by a 10 second unloading to 0 N. A 15 × 10 indentation matrix was performed on each sample, resulting in 150 individual measurements on each sample. Spacing between indents was 50 µm in both x and y directions. The elastic modulus was calculated from the unloading portion of the load-displacement curve using the Oliver-Pharr method^27^. A Poisson’s ratio of 0.21 was taken for WC-Co in order to calculate the elastic modulus^28^.

The microscale tribological behavior of the nanocomposite was investigated via microscale scratch tests using an MTS Nano Indenter XP in scratch mode. Scratch tests were performed on the polished cross-sections with a Berkovich tip, where the scratch direction was along one of the edges of the Berkovich tip. The polished cross-sections of the WC-Co, WC-Co-2ND, and WC-Co-10ND samples had a surface roughness of ~11.5–12.5 nm, as determined by atomic force microscopy (AFM). The WC-Co-5ND polished cross-section had a surface roughness of ~25 nm, despite identical polishing procedures. The scratches were performed with a constant normal load of 200 mN, a scratch speed of 1.0 µm/s, and a scratch length of 30 µm. Lateral forces were measured during the scratch test to calculate coefficient of friction, which is the ratio of lateral to normal forces. The residual depths of the scratch grooves were profiled using a Dimension 3100 AFM (Digital Instruments, Veeco Metrology Group, Santa Barbara, CA). The AFM profiling was done using a scan rate of 0.10 Hz and a tip velocity of 10 µm/s. The 3-D profiles obtained by the AFM were then sectioned into 2-D profiles in order to measure the cross-sectional area of the scratch groove. The cross-sectional area was multiplied by the known scratch length of 30 µm to calculate the volume lost during the scratch test (i.e., wear volume). AFM profiling was also used to characterize the average surface roughness (Ra) of the polished samples. The average of 5 scratch tests was utilized to calculate values and the error bars reported represent one standard deviation.

Macroscale reciprocating dry sliding wear tests were conducted using a NeoPlus RFW-160 (Daejeon, South Korea) high temperature tribometer in order to assess the macroscale coefficient of friction (CoF) and wear resistance of the coatings. Silicon nitride balls with a diameter of 5 mm were used as the counter surface; a new ball was used for each test. The wear tests were run for 60 min utilizing a normal load of 50 N, a stroke length of 10 mm, and a frequency of 2 Hz, which corresponds to 7200 cycles and a total sliding distance of 144 m. Wear tests were conducted at room temperature (RT, ~25 °C) under ambient conditions. Samples were lightly polished with SiC abrasive paper prior to wear tests to remove residue from the sintering dies, resulting in an average roughness (R_a_) of 4–5 µm prior to wear testing, as measured by a 3D laser microscope. The depth and volume profiles of the wear tracks were obtained using a Keyence VK-X200 (Osaka, Japan) 3D laser microscope in order to measure the wear volume and thereby gauge the wear resistance of the coatings. Wear volume was obtained by calculating the average wear track cross sectional area of over 100 cross sections, and then multiplying it by the stroke length. Reported wear volumes and CoF values are averages derived from at least three wear tests. Mounted cross sections were ground using SiC abrasive papers and diamond slurry using the same procedure that was utilized for preparing surfaces for nanoindentation.

### Structural and Chemical Characterization

The starting ND powders were characterized by transmission electron microscopy (TEM) using a JEOL 2500 TEM operating at 200 keV in order to assess the size of the ND particles. ND powders were dispersed into a methanol solution via ultrasonication and then a drop of the dispersion was deposited onto a lacy carbon TEM grid. An FEI Scios dual-beam focused ion beam – scanning electron microscope (FIB-SEM) was utilized to characterize the powders, consolidated microstructures, and wear track morphologies. WC particle sizes were analyzed using Image J software. Energy dispersive spectroscopy (EDS) analysis was conducted on an FEI Scios FIB-SEM equipped with an Oxford EDS system to characterize the elemental composition of phases present on wear tracks. Phases in the sintered samples and corresponding wear tracks were analyzed using a Scintag XDS2000 X-ray diffractometer. The operating voltage and current were 45 kV and 40 mA, respectively. Surface chemistry of the sintered samples and corresponding wear tracks was analyzed via X-ray photoelectron spectroscopy (XPS) using a Thermo Scientific K-alpha XPS. The density of sintered samples was measured using the Archimedes method, and relative densities were calculated based on the known densities of WC-Co and ND. WC particle sizes were measured using Image J software; averages presented are based on over 100 measurements.

## Electronic supplementary material

Supplementary Information
