# Supplementary material for: Synthesis and Multi Scale Tribological Behavior of WC-Co/Nanodiamond Nanocomposites
Source: Sci Rep. 2017 Aug 1;7:7060. doi: 10.1038/s41598-017-07324-3 (PMC5539160; doi:10.1038/s41598-017-07324-3)
Supplement: Supplementary file 1 — Supplementary Information [file 41598_2017_7324_MOESM1_ESM.pdf]

Supporting Information for

Synthesis and Multi Scale Tribological Behavior of WC-Co/Nanodiamond Nanocomposites

Andy Nieto[a,c], Lin Jiang[a,b], Jaekang Kim[c,d], Dae-Eun Kim[c,d*], Julie M. Schoenung[a,b*]

[a] Department of Chemical Engineering and Materials Science,
University of California-Davis, Davis, CA, 95616, USA

[b] Department of Chemical Engineering and Materials Science,
University of California-Irvine, Irvine, CA, 92697, USA

[c] Center for Nano-Wear,
Yonsei University, Seoul 120-749, Republic of Korea

[d] Department of Mechanical Engineering,
Yonsei University, Seoul 120-749, Republic of Korea

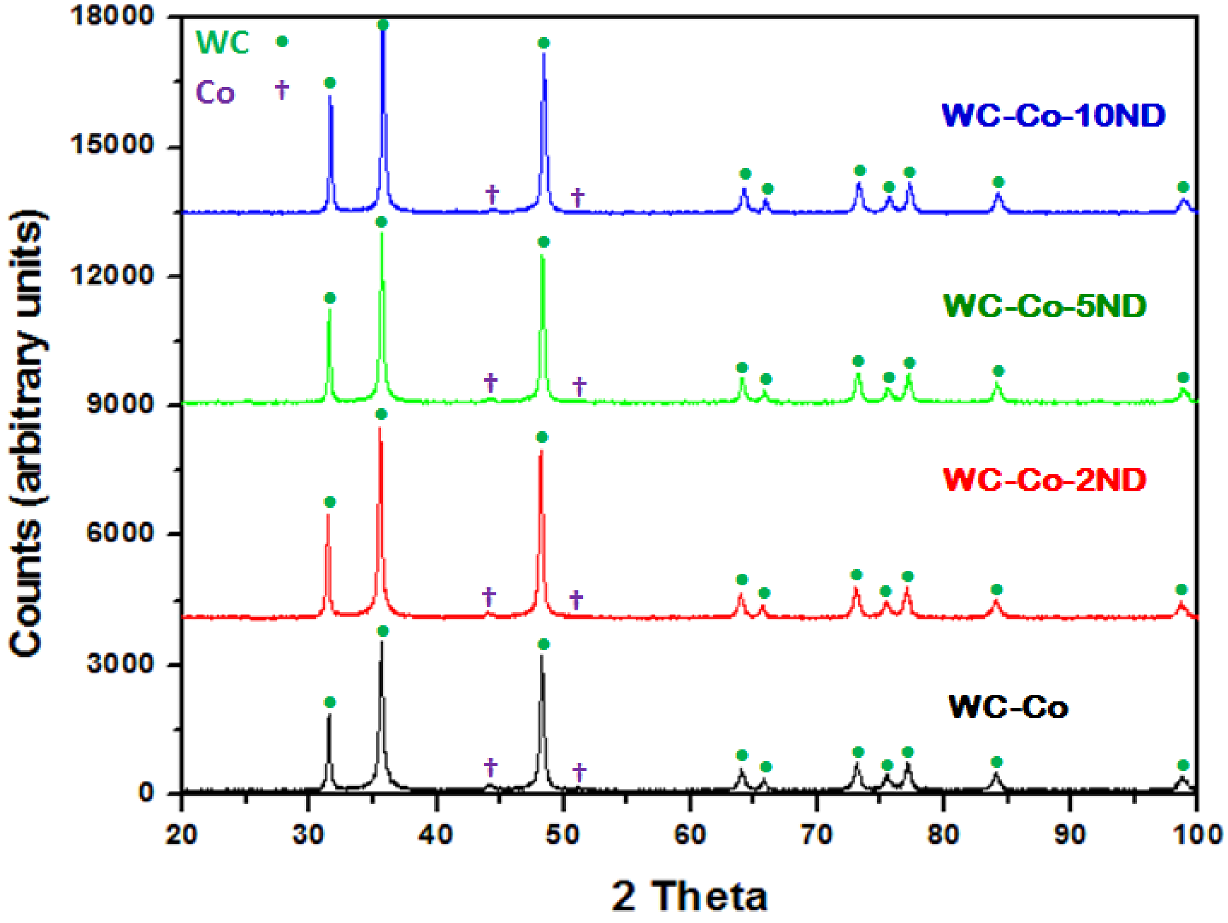

Figure S1: X-ray diffraction (XRD) patterns for spark plasma sintered (SPS) WC-Co and composites.

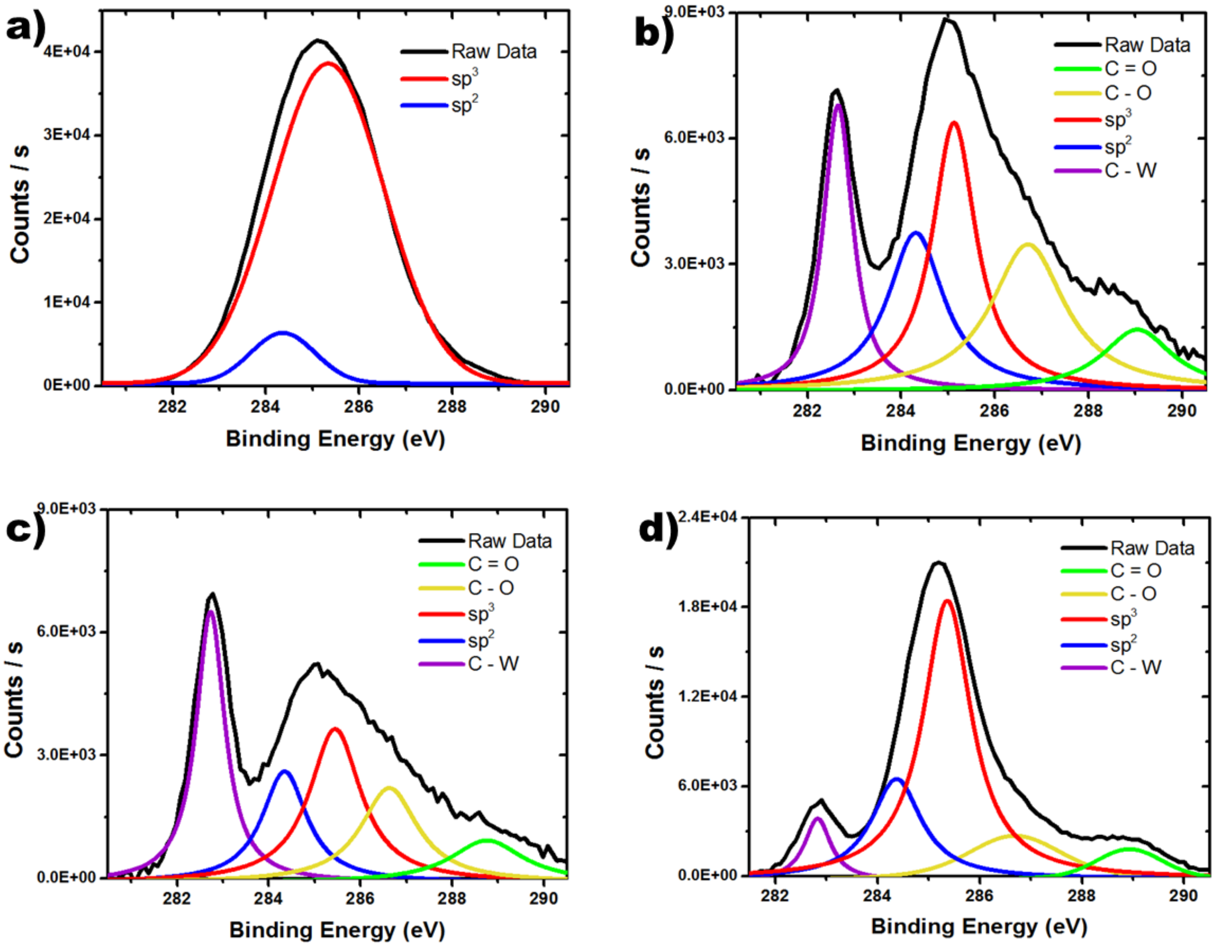

Figure S2: a) X-ray photoelectron spectra (XPS) for: a) starting nanodiamond (ND) powder, b) SPS WC-Co-2ND sample, c) SPS WC-Co-5ND sample, d) WC-Co-10ND sample

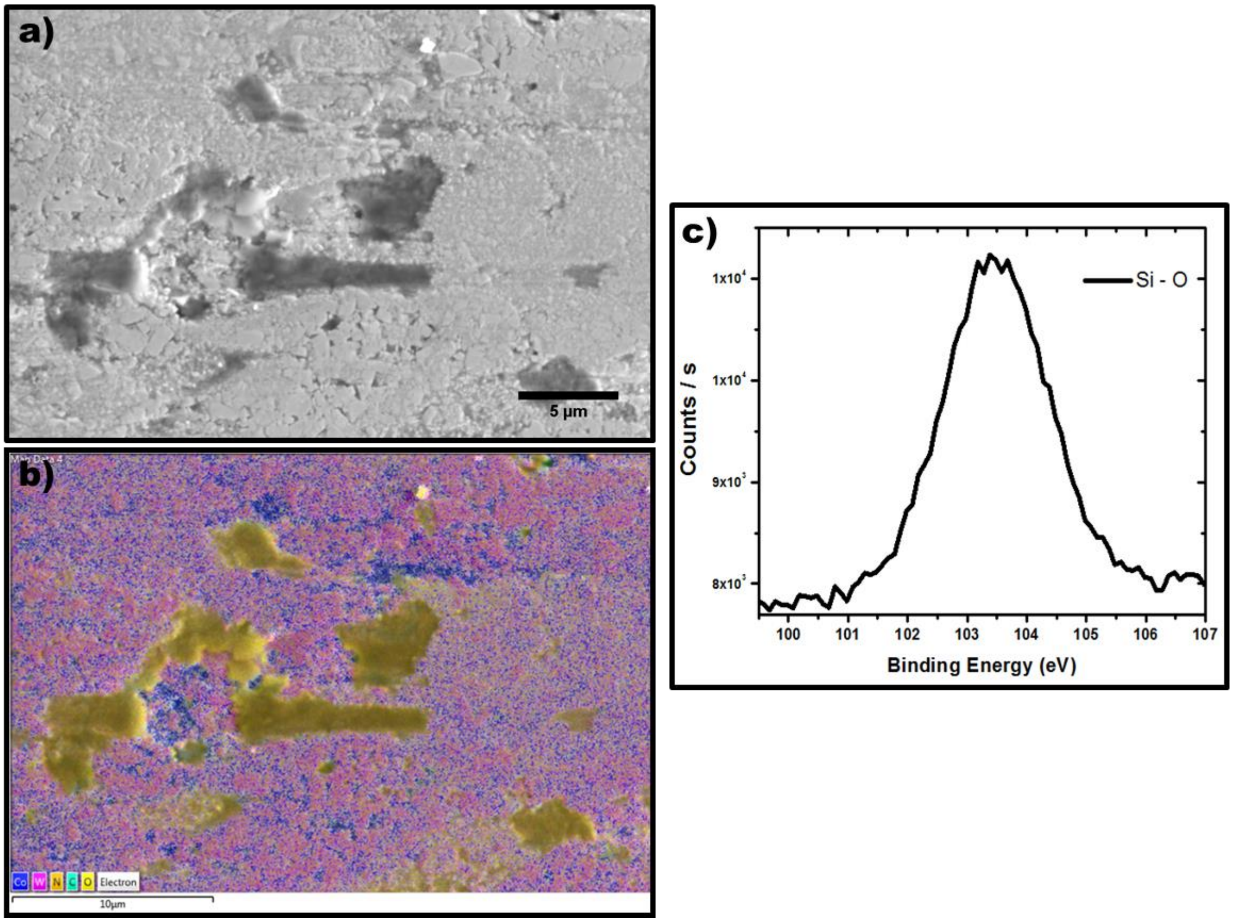

Figure S3: a) Scanning electron micrograph (SEM) of tribofilms on WC-Co-2ND wear tracks, b) Energy dispersive spectra (EDS) map of silica tribofilm on WC-Co-2ND samples shown in a), c) XPS of S-O bond in WC-Co-10ND

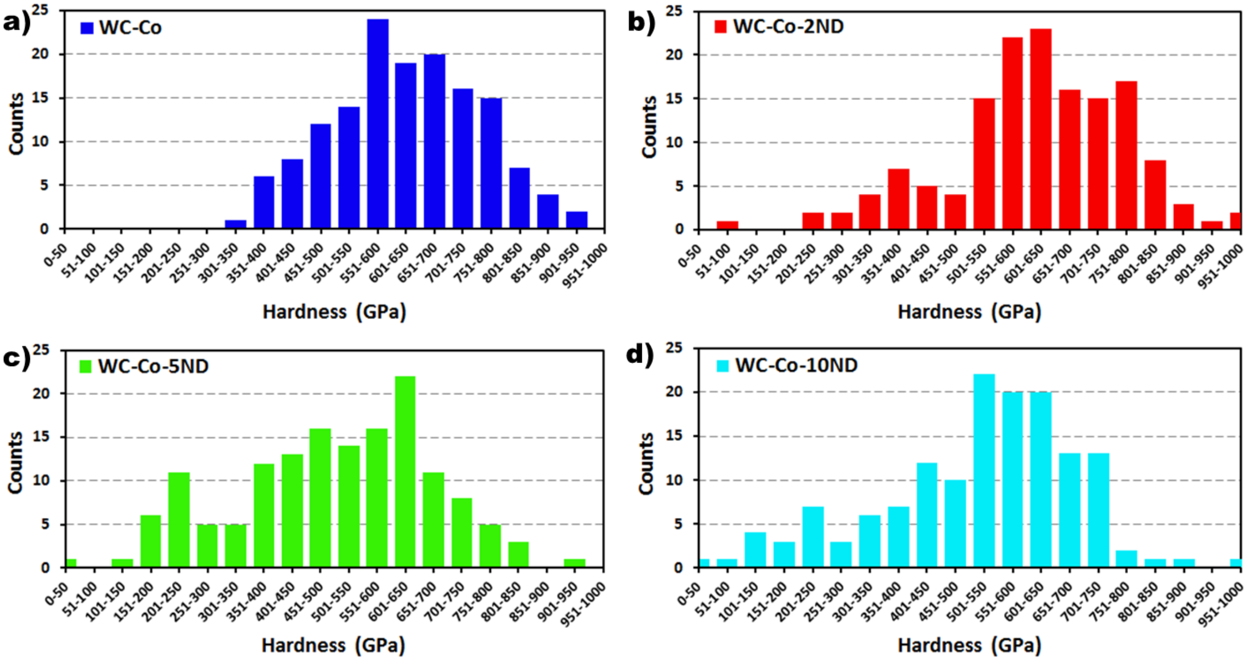

Figure S4: Distributions in elastic modulus measured via nanoindentation: a) WC-Co, b) WC-Co-2ND, c) WC-Co-5ND, d)WC-Co-10ND
